# Valorization of lignin and cellulose in acid-steam-exploded corn stover by a moderate alkaline ethanol post-treatment based on an integrated biorefinery concept

**DOI:** 10.1186/s13068-016-0656-1

**Published:** 2016-11-08

**Authors:** Sheng Yang, Yue Zhang, Wen Yue, Wei Wang, Yun-Yan Wang, Tong-Qi Yuan, Run-Cang Sun

**Affiliations:** 1 Beijing Key Laboratory of Lignocellulosic Chemistry, Beijing Forestry University, Beijing, 100083 People’s Republic of China; 2 Department of Chemical Engineering, University of Florida, Gainesville, FL 32603 USA; 3 Textile Application, Research & Development Center, Novozymes (China) Investment Co. Ltd, Beijing, 100085 People’s Republic of China; 4 Department of Bioproducts and Biosystems Engineering, University of Minnesota, Saint Paul, MN 55108-6130 USA

**Keywords:** Corn stover, Acid-steam-explosion, Alcoholic NaOH, Post-treatment, Enzymatic hydrolysis, Lignin, Lignin-phenol-formaldehyde resin, Biorefinery

## Abstract

**Background:**

Due to the unsustainable consumption of fossil resources, great efforts have been made to convert lignocellulose into bioethanol and commodity organic compounds through biological methods. The conversion of cellulose is impeded by the compactness of plant cell wall matrix and crystalline structure of the native cellulose. Therefore, appropriate pretreatment and even post-treatment are indispensable to overcome this problem. Additionally, an adequate utilization of coproduct lignin will be important for improving the economic viability of modern biorefinery industries.

**Results:**

The effectiveness of moderate alkaline ethanol post-treatment on the bioconversion efficiency of cellulose in the acid-steam-exploded corn stover was investigated in this study. Results showed that an increase of the alcoholic sodium hydroxide (NaOH) concentration from 0.05 to 4% led to a decrease in the lignin content in the post-treated samples from 32.8 to 10.7%, while the cellulose digestibility consequently increased. The cellulose conversion of the 4% alcoholic NaOH integrally treated corn stover reached up to 99.3% after 72 h, which was significantly higher than that of the acid steam exploded corn stover without post-treatment (57.3%). In addition to the decrease in lignin content, an expansion of cellulose I lattice induced by the 4% alcoholic NaOH post-treatment played a significant role in promoting the enzymatic hydrolysis of corn stover. More importantly, the lignin fraction (AL) released during the 4% alcoholic NaOH post-treatment and the lignin-rich residue (EHR) remained after the enzymatic hydrolysis of the 4% alcoholic NaOH post-treated acid-steam-exploded corn stover were employed to synthesize lignin-phenol-formaldehyde (LPF) resins. The plywoods prepared with the resins exhibit satisfactory performances.

**Conclusions:**

An alkaline ethanol system with an appropriate NaOH concentration could improve the removal of lignin and modification of the crystalline structure of cellulose in acid-steam-exploded corn stover, and consequently significantly improve the conversion of cellulose through enzymatic hydrolysis for biofuel production. The lignin fractions obtained as byproducts could be applied in high performance LPF resin preparation. The proposed model for the integral valorization of corn stover in this study is worth of popularization.

## Background

The depletion of fossil fuel resources and ever-growing greenhouse gas has resulted in an increasing worldwide interest in alternative nonfossil-based sources of energy [1, 2]. At present, governments across the globe are committed to identifying new clean energies to power the world’s future with sustainable supply capacity and low- or zero-carbon emissions. Bioethanol, a fuel produced from lignocellulosic biomass, is largely considered a favorable potential alternative to fossil fuels [1, 2]. The conversion of lignocellulosic biomass to bioethanol involves the hydrolysis of cellulose to monosaccharides, and then the fermentation of these sugars into ethanol. Enzymatic hydrolysis is a key step in the ethanol production from low-cost lignocellulosic materials [3, 4]. Due to compactness of lignocellulose matrix and the crystalline structure of the native cellulose [5, 6], pretreatment is necessary to disrupt the recalcitrant matrix to make the cellulose more accessible to the enzymes, which convert carbohydrate polymers into fermentable sugars [7]. These pretreatment technologies can be classified into biological, physical, chemical, and physicochemical methods, including dilute acid, steam explosion, hot-compressed water, organosolv, ammonia fiber explosion, and aqueous lime or alkali pretreatments [8–13]. All of the pretreatment methods have limitations; therefore, the synergistic benefits of combined methods should be considered [14].

Acid steam explosion (ASE) is one of the most extensively studied pretreatment methods, which opens the compact lignocellulosic plant cell walls and effectively removes most of the hemicelluloses, consequently increases the efficiency of cellulose hydrolysis [15, 16]. The acid-steam-exploded residues are mainly composed of cellulose and lignin. Particularly, it is well known that lignin limits the digestibility of lignocelluloses [17–19]. Therefore, a delignification process is essential for the biomass before it is subjected to enzymatic hydrolysis. It should be noted that the lignin in the corn stover has been partially degraded and deposited on the surface of the treated corn stover during the ASE process [20]. Thus, just relatively mild post-treatment may be needed to eliminate the remaining lignin fraction in the lignocellulosic biomass for promoting the subsequent enzymatic hydrolysis. However, the reports about such study are rare.

NaOH solution has been reviewed to be efficient in dissolving lignin, and causes lignocellulosic biomass to swell leading to an increase in the internal surface area under relatively mild conditions, thus it enhances the enzymatic hydrolysis of lignocellulosic biomass [21, 22]. Ethanol has been widely used in lignocelluloses treatment as a solvent for increasing the dissolution of the degraded lignin fractions [23]. After the removal of ethanol, the obtained high concentration lignin solution can be directly used to prepare other chemicals or materials without any further treatments. In fact, in addition to the high concentration lignin solution, a large amount of lignin-rich residue is also recovered after enzymatic hydrolysis and fermentation. From the economic point of view, many cellulosic ethanol demonstration plants have attempted to integrate their key technologies into processes including the pretreatment of raw materials, hydrolysis, fermentation, distillation, and waste treatment [24]. For these reasons, the effectiveness of the mild alkaline ethanol post-treatment of the acid steam exploded materials in terms of an integrated biorefinery process would be a quite worthwhile research object.

The main purpose of this study was to investigate the effectiveness of the moderate alkaline ethanol post-treatment on the enzyme hydrolysis efficiency of cellulose in ASE technology. The post-treated samples obtained were subjected to composition analysis and characterized by Fourier-transform infrared (FT-IR), solid-state cross polarization/magic angle spinning (CP/MAS) ^13^C-NMR spectroscopy, and X-ray diffraction (XRD). The analysis results were used to evaluate the factors affecting enzymatic hydrolysis. The lignin samples obtained from the moderate alkaline ethanol post-treatment were analyzed and applied to synthesize lignin-phenol-formaldehyde (LPF) resin, and the applicability of lignin-rich residue in LPF resin synthesis was also evaluated. A potential model for integral valorization of corn stover will be proposed in this study.

## Results and discussion

### Compositional analysis

The acid-steam-exploded corn stover was post-treated with alkaline ethanol with different NaOH concentrations, and an acid sodium chlorite post-treated acid-steam-exploded corn stover was also obtained as a comparison (an extensively delignified sample). In Table 1, all the samples have been identified by the different post-treatment methods used. The compositions of untreated corn stover, acid-steam-exploded corn stover, the post-treated samples, and the solid yields of each post-treated samples are given in Table 1. As compared with the composition of untreated corn stover (36.5% cellulose, 22.1% hemicelluloses, and 18.8% lignin), the ratio of relative content of cellulose to lignin slightly decreased in the acid-steam-exploded materials (Sample 1) due to the significant degradation of hemicellulose during the steam-explosion process. In addition, this phenomenon also implied that the compact matrix of plant cell wall has been significantly modified and some of the cellulose has been degraded during the ASE treatment. The accessibility of cellulase to cellulose could be significantly improved, and the conversion rate of cellulose should also increase [25]. However, as shown in Table 1, lignin, which limits the digestibility of lignocelluloses, remains in the acid-steam-exposed corn stover in a relative content of 32.8%. In the integrated corn stovers treatment, the ratio of cellulose to lignin increases dramatically as the NaOH concentration increases. Generally, the gradual removal of lignin was accompanied with the increase of conversion of cellulose [26]. At 4% concentration of NaOH (Sample 4), the relative content of cellulose reached up to 84.0% with only 10.7% lignin remained in the post-treated sample. To this effect, alkaline ethanol post-treatment under the mild conditions was proved to be a very effective delignification method. Sample 5, which was post-treated with acid sodium chlorite, had the lowest lignin content (3.0%) and highest cellulose content (89.2%) among the five samples. Such treatment may result in the highest cellulose conversion rate in this study, yet other factors may also contribute to the cellulose conversion result, and they need to be considered.

**Table 1:** Compositional analysis of the untreated, acid-steam-exploded and integrally treated corn stovers

Samples	Treatment methods	Solid yields	Cellulose	Hemicelluloses	Lignin	Others
Corn stover	Untreated	–	36.5 (2.1)	22.1 (0.9)	18.8 (1.7)	22.6 (1.3)
1	Acid steam explosion	–	55.6 (3.5)^a^	5.7 (0.7)	32.8 (2.6)	5.9 (0.9)
2	Acid steam explosion followed by a 0.05% alcoholic NaOH post-treatment	78.1	71.1 (2.8)	6.4 (0.6)	19.1 (3.4)	3.4 (0.6)
3	Acid steam explosion followed by a 0.5% alcoholic NaOH post-treatment	70.3	78.9 (3.3)	4.2 (0.6)	15.9 (3.2)	1.0 (0.1)
4	Acid steam explosion followed by a 4% alcoholic NaOH post-treatment	65.4	84.0 (4.4)	4.1 (0.7)	10.7 (1.7)	1.2 (0.8)
5	Acid steam explosion followed by a NaClO_2_ post-treatment	59.6	89.2 (2.5)	7.5 (1.1)	3.0 (0.4)	0.3 (0.1)

^a^The value in parenthesis is standard deviation

The relative proportions of hemicelluloses of the five samples listed in Table 1 (4.1–7.5%) did not vary much from one another as compared to the changes in cellulose and lignin. Therefore, the influence of hemicelluloses on sugar yield can be ignored in this work. It should be mentioned that only xylose in hemicelluloses was lost during the post-treatment process. A series of alkaline ethanol with different NaOH concentrations were processed to obtain various degrees of delignification, but only three are displayed in Table 1. It should be noted that lignin content presented a gradient decrease (32.8–10.7%) within the four post-treated samples (Sample 1–4) which may be an ideal series to investigate the effects of lignin content on the enzymatic hydrolysis of cellulose in the subsequent studies.

### FT-IR spectra

**Fig. 1:**
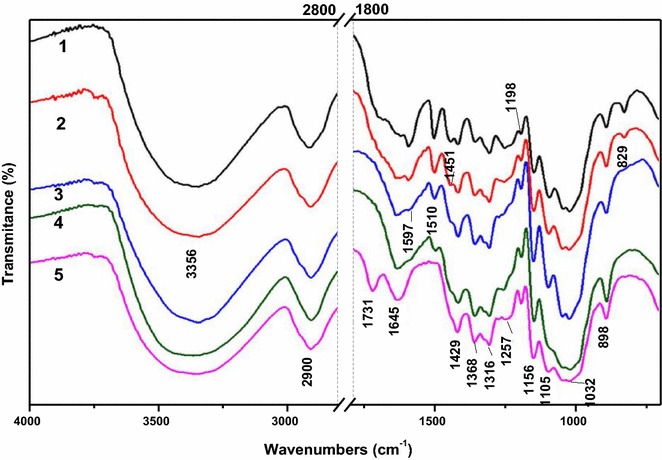
FT-IR spectra of the acid-steam-exploded and the integrally treated corn stovers

The FT-IR spectra of the acid-steam-exploded corn stover and the post-treated samples are shown in Fig. 1. The bands at 1597, 1510, and 1429 cm^−1^ are corresponding to aromatic skeletal vibrations, and C–H deformation combined with aromatic ring vibration was found at 1451 cm^−1^ [27, 28]. In Samples 1–4, the four characteristic absorption peaks of lignin became gradually weaker as the NaOH concentration increased. These four peaks almost disappeared in Sample 5, which was post-treated with acid sodium chlorite. Correspondingly, gradual increase of intensities of the bands at 1198 and 1156 cm^−1^ corresponding to O–H stretching and C–O–C vibrations at *β*-glucosidic linkages in cellulose were observed. These phenomena altogether indicated that the relative contents of cellulose in Samples 1–5 increased due to the increasing removal of lignin. The FT-IR results obtained above were all in reasonable agreement with the compositional analysis (Table 1). Additionally, a new peak at 1731 cm^−1^, which is assigned to non-conjugated carbonyl groups [29], appeared in the spectrum of Sample 5, which was probably that the oxidation of the cellulose occurred during the process of acid sodium chloride delignification.

### CP/MAS ^13^C-NMR spectra

**Fig. 2:**
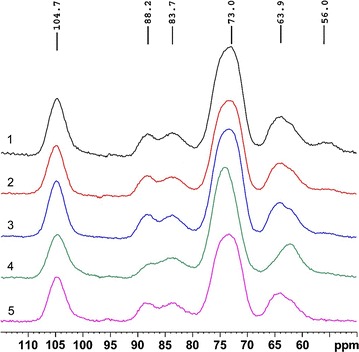
CP/MAS ^13^C-NMR spectra of the acid-steam-exploded and the integrally treated corn stovers

To further investigate the structural characteristics of the acid-steam-exploded corn stover and the post-treated corn stovers, all the samples were subjected to CP/MAS ^13^C-NMR spectroscopy analysis. The corresponding spectra are depicted in Fig. 2. The region between 55 and 110 ppm was dominated by strong signals assigned mostly to various cellulosic carbons, and the sharp signal at 104.7 ppm is attributed to the ordered cellulose C-1. The signals at 68–80 ppm are due to cellulose C-2, C-3, and C-5 [30, 31]. The relative intensity of the signal at 56.0 ppm, which corresponds to the methoxyl groups (–OCH_3_) of aromatic moieties in lignin, decreased as the NaOH concentration increased until nearly disappearing entirely in the spectrum of Sample 5. Analogous results have been reflected in the compositional and FT-IR analyses discussed above.

Obvious signals for amorphous and crystalline carbons of cellulose could be detected. The chemical shifts at 88.2 and 63.9 ppm are ascribed to crystalline cellulose C-4 and C-6 carbons, respectively. The signal at 83.7 ppm is due to amorphous cellulose C-4 carbons, and the signal at 62.2 ppm is attributed to amorphous cellulose C-6 [32]. In Sample 4, which was delignified with 4% alcoholic NaOH, the intensity of the signal at 88.2 ppm decreased and increased at 83.7 ppm, respectively. The peak at 63.9 ppm seemingly disappeared and shifted to 62.2 ppm in Sample 4, but the peaks at 63.9 and 62.2 ppm coexisted in Samples 1–3 and 5. This indicated that part of the cellulose structure of Sample 4 transformed from the original crystalline cellulose into amorphous cellulose. The amorphous cellulose is more vulnerable to cellulose enzymes and thus favors glucan conversion [33]. It should be noted that the signal at 63.9 ppm in the spectrum of Sample 4 should not be completely disappeared. The intensity of this signal in Sample 4 was weak and overlapped with other signal due to the poor resolution of the NMR method used. In Sample 5, both of the crystalline signals 88.2 and 63.9 ppm have not shifted. It seems that the crystalline domain of cellulose was not disrupted by acid sodium chlorite post-treatment.

### XRD analysis

**Fig. 3:**
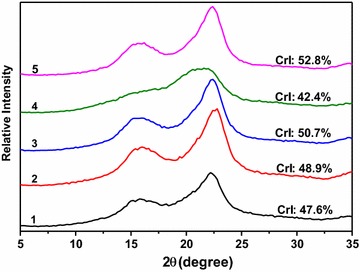
X-ray diffractograms of the acid-steam-exploded and the integrally treated corn stovers

Cellulose crystal structure is considered one of the major substrate properties influencing biomass enzymatic digestibility [33]. In this study, the acid-steam-exploded corn stover and the post-treated samples were examined by XRD to gain insight into the potential structural features affecting cellulose hydrolysis (Fig. 3). The crystallinity index (*CrI*) values of Samples 1–5 were calculated to be 47.6, 48.9, 50.7, 42.4, and 52.8%, respectively. The slight increase of *CrI* in Samples 1–3 was due to the gradual removal of amorphous lignin and hemicelluloses with alkaline ethanol. While for Sample 5, it was due to the removal of lignin with acid sodium chlorite. All these four samples exhibited typical diffraction patterns of cellulose I, the main peak of which lies near 22.5° and the secondary broad peak at ~16.0°. In fact, the broad peak at ~16.0° should be consisted of two small peaks at 15.2° and 16.8°, but the XRD analysis conditions used make it hard to be distinguished as reported by Trache et al. [34]. In Sample 4, conversely, the shape of diffractogram and trend of *CrI* differed notably as compared to other samples. The main peak around 22.5° was weakened and shifted slightly to a lower angle, and there was no peak at 12.1° (one of the typical diffraction patterns of cellulose II). These results confirmed that the cellulose in Sample 4 was in a transition status from cellulose I to cellulose II, and that an expansion of cellulose I lattice occurred. It has been reported that this benefited the conversion of cellulose to glucose [35]. It is important to note, however, that 4% aqueous NaOH post-treatment alone under similar conditions would not lead to the same results [35]. It may be due to that the solubility of lignin in 4% alcoholic NaOH was higher than that in 4% aqueous NaOH. Thus, the exposed surface area of cellulose in 4% alcoholic NaOH was more than that in 4% aqueous NaOH, and the cellulose I lattice was easy to be expanded under such moderate condition.

### Enzymatic hydrolysis

**Fig. 4:**
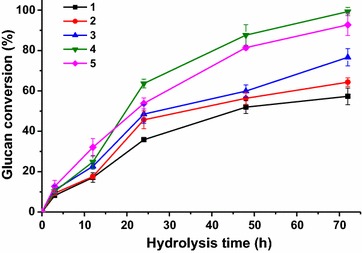
Enzymatic hydrolysis of the acid-steam-exploded and the integrally treated corn stovers

To evaluate the effects of different post-treatment conditions on glucose yield, the enzymatic hydrolyses of the acid-steam-exploded corn stover and the post-treated samples were comparatively studied as shown in Fig. 4. Cellulose digestibility was 57.3% for the only acid-steam-exploded material (Sample 1) within 72 h enzymatic hydrolysis, and for the post-treated Samples 2, 3 and 5, the glucose yield was 64.3, 76.7, and 92.7%, respectively. The corresponding lignin contents of Samples 1–3 and 5 were 32.8, 19.1, 15.9, and 3.0%, respectively. These results indicated that the efficiency of enzymatic hydrolysis was significantly affected by the remaining lignin content, and it increased as the lignin content decreased. In general, cellulose conversion is known to decrease as the *CrI* of cellulose increases, and it has been already reported that the *CrI* is related to cellulose content or alfa-cellulose [31]. However, this phenomenon was not observed in the data of Samples 1–3 or 5 in this study. This indicated that the content of lignin in the lignocelluloses is also a very important factor affecting the rate of enzymatic hydrolysis except for the crystallinity of the material. As the lignin content decreased in the sample, the rate of the enzymatic hydrolysis increased. In addition, the results of compositional analysis indicated that the cellulose in the acid-steam-exploded corn stovers was not obviously removed during alkaline ethanol post-treatment. Most of the alfa-cellulose remained in the post-treated samples, and its relative content increased as a result of the removal of amorphous components (lignin and hemicelluloses). Only a slight removal of non-crystalline cellulose may be occurred during the process.

It was interesting that the cellulose digestibility of Sample 4 reached a maximum of 99.3% after 72 h, higher than that (92.7%) of Sample 5 despite its relatively high lignin content (10.7%). XRD analysis showed that the *CrI* of Sample 4 was 42.4% and the cellulose was in a transition status from cellulose I to cellulose II, which implied that an apparent change in *CrI* (especially cellulose lattice transformation) had an important impact on the enzymatic hydrolysis of cellulose in addition to the lignin content. It has been reported that along with the transformation of cellulose I to cellulose II [36], the expansion of cellulose I lattice promotes the enzymatic hydrolysis of cellulose [35, 37, 38].

Besides, it was found that Sample 4 had a higher enzymatic hydrolysis rate than those of other samples after 24 h. It was hypothesized that lignin was the main obstacle to cellulose degradation within the first 24 h when the degraded substrate was mainly amorphous cellulose. This is the reason why the initial rate of Sample 4 was lower than that of Sample 5 although more amorphous fractions were found in Sample 4. In fact, lignin is also a main factor for the increase of the material recalcitrance and decrease the enzymatic hydrolysis rate [39]. After 24 h of saccharification, the crystalline structure of cellulose in turn became the main obstacle to cellulose hydrolysis. The amorphous structure of the cellulose or cellulose II in Sample 4 made it more accessible to cellulase than those of other samples. Thus, the enzymatic hydrolysis rate of Sample 4 was higher than those of other samples after 24 h, and the glucose yield of Sample 4 reached up to 99.3% in a relatively short period of hydrolysis. Overall, an alkaline ethanol system with appropriate NaOH concentration that is able to effectively remove lignin and change the crystalline structure of cellulose under moderate conditions represents a promising approach to modern biorefinery.

### Characterization of lignins

The characterization of lignins obtained could facilitate the understanding of treatment process and reasonable utilization of the byproducts. It could be found that approximately 60% of lignin in the acid-steam-exploded corn stover was removed during 4% alcoholic NaOH post-treatment. The compositions of the lignin fraction (AL) released during the 4% alcoholic NaOH post-treatment and lignin-rich residue (EHR) remained after the enzymatic hydrolysis of the 4% alcoholic NaOH post-treated acid-steam-exploded corn stover are listed in Table 2. The relatively high purity (more than 80%) of AL made this isolated lignin a good material for subsequent utilization. The utilization of AL in LPF resin preparation could be regarded as a component part of the integrated biorefinery of corn stover. The realization of this approach could help to overcome the limitation of byproducts dispose on economic benefit and production scale of corn stover biorefinery. Meanwhile, it could indirectly promote the extension of biorefinery industry toward energy and advanced materials. EHR was obtained via centrifugation followed by freeze-drying. It could be observed that the content of remained carbohydrates (3.82%) in EHR was very low. The relatively high content of lignin in EHR (75.43%) made it as an ideal material for LPF resin preparation. The remained cellulase, which was composed by protein, was also ideal material for wood adhesive production like lignin dose [40]. Thus, the EHR obtained was also potential material for LPF resin synthesis. The application of this hydrolysis residue could help to realize the whole component utilization of corn stover.

**Table 2:** Compositional analysis of the isolated lignin fraction from the 4% alcoholic NaOH treatment producer and the corresponding enzymatic hydrolysis residue

Samples	Lignin content (%)		Carbohydrate content (%)						
	AIL	ASL	Rha	Ara	Gal	Glc	Man	Xyl	GlcA/GalA
AL	78.83 (0.86)^b^	3.32 (0.47)	0.23 (0.06)	–^a^	–	0.52 (0.11)	–	4.62 (0.14)	–
EHR	71.83 (0.48)	3.60 (0.75)	–	–	–	2.64 (0.14)	–	0.86 (0.02)	0.32/– (0.02/–)

*AL* alkaline lignin obtained from the 4% alcoholic NaOH post-treatment producer, *EHR* lignin-rich residue remained after enzymatic hydrolysis of the 4% alcoholic NaOH post-treated acid-steam-exploded corn stover, *AIL* acid insoluble lignin, *ASL* acid soluble lignin, *Rha* rhamnose, *Ara* arabinose, *Gal* galactose, *Glc* glucose, *Man* mannose, *Xyl* xylose, *GlcA* glucuronic acid, *GlaA* galacturonic acid

^a^Not detected

^b^The value in parenthesis is standard deviation

**Fig. 5:**
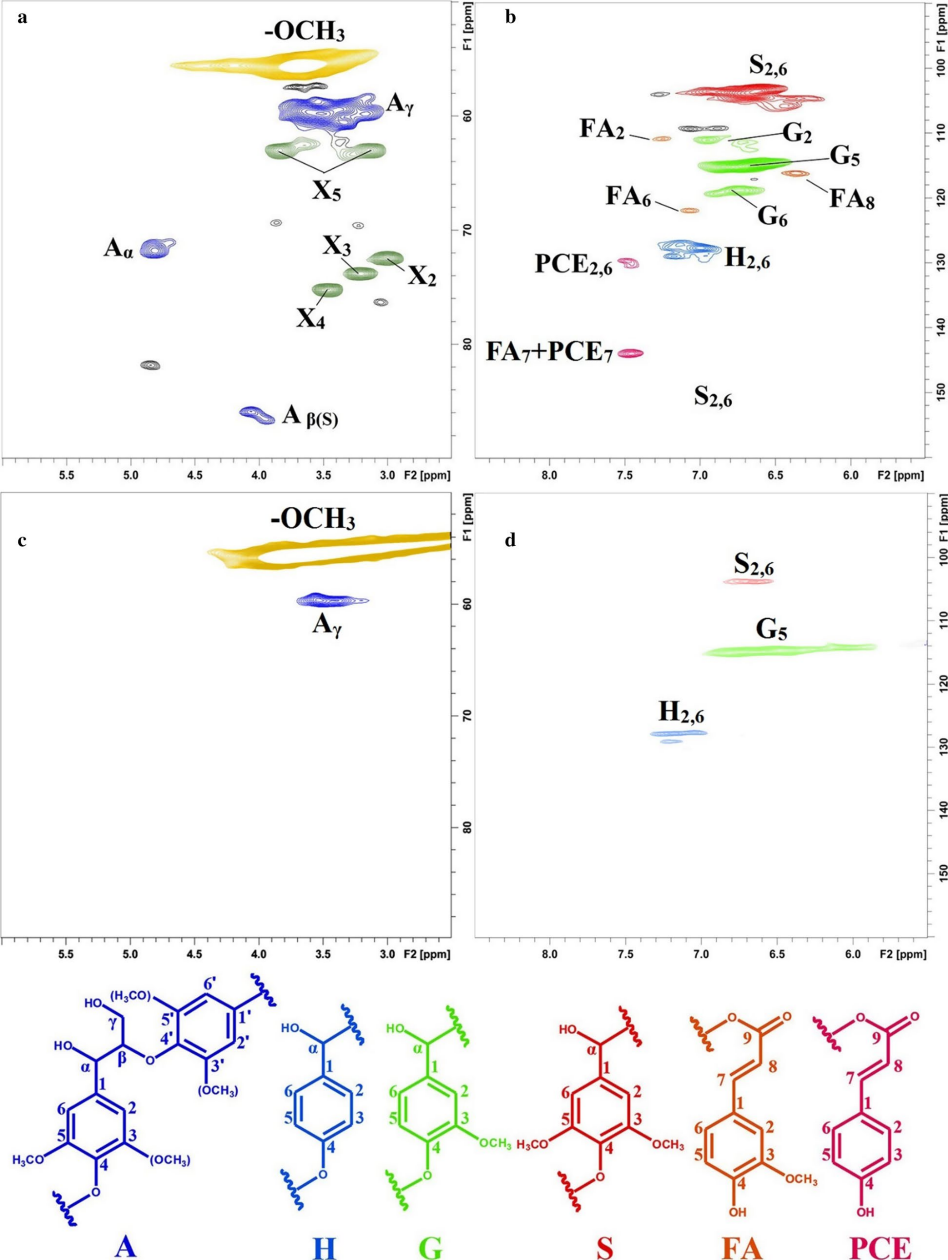
2D ^13^C-^1^H correlation (HSQC) spectra of AL and purified EHR (**a** Aliphatic region of AL; **b** Aromatic region of AL; **c** Aliphatic region of purified EHR; **d** Aromatic region of purified EHR. Key structural details of lignin: (*A*) *β*-*O*-4′ aryl ether linkages; (*H*) *p*-hydroxyphenyl units; (*G*) guaiacyl units; (*S*) syringyl units; (*PCE*) *p*-coumarates; (*FA*) ferulates; (*X*) *β*-d-Xylp)

The two dimensional heteronuclear single-quantum correlation (2D HSQC) spectra of AL and purified EHR are given in Fig. 5 and the spectra were annotated with peak assignments based on previous publications [41, 42]. The structures of the identified lignin sub-units in the two lignins are also depicted in Fig. 5. In the side-chain region of the 2D HSQC spectra of AL, cross-signals of methoxyls (*δ*_C_/*δ*_H_ 55.9/3.73) and *β*-*O*-4′ aryl ether linkages were the most prominent. The C_*β*_–H_*β*_ correlation in *β*-*O*-4′ substructures (structure A) were observed at *δ*_C_/*δ*_H_ 72.2/4.86. The C_*β*_–H_*β*_ correlations corresponding to the Syringyl-type *β*-*O*-4′ substructures could be seen at *δ*_C_/*δ*_H_ 85.8/4.12. It could be found that the intensities of the signals originated from *β*-*O*-4′ substructures in the spectrum of AL were significantly lower than those of the native lignin from the untreated corn stover as detected by our previous work [43]. This indicated that most of the *β*-*O*-4′ linkages in lignin macromolecule have been cleaved during the integrated treatment process. The signals of other substructures found in the spectrum of native lignin in untreated corn stover, such as resinol, phenylcoumaran, and spirodienone, were also absent in the spectrum of AL. The results indicated that lignin in acid-steam-exploded corn stover has been partially degraded and the moderate post-treatment conditions used in this study were competent for removing the lignins. However, it should be noted that the linkages between lignin units were hard to cleave during alkaline ethanol post-treatment in this study. The cleavage of *β*-*O*-4′ and other side-chain linkages should mainly occur during ASE treatment [44].

Weak signals of *β*-*O*-4′ and other side-chain linkages implied that the AL possessed low molecular weight and relatively high phenolic hydroxyl content. Therefore, AL should be a precious phenolic material for LPF resin synthesis. Furthermore, in the 2D HSQC spectra of AL, the signals arising from *β*-d-Xylp were evidently noted, with its C_2_–H_2_, C_3_–H_3_, C_4_–H_4_, and C_5_–C_5_ correlations at *δ*_C_/*δ*_H_ 72.7/2.89, 73.6/3.22, 75.5/3.59, and 62.6/3.40 and 3.72, respectively. This implied that a certain amount of xylans still remained in AL, which was well consistent with the result obtained in carbohydrate analysis of AL (Table 2). Xylans were mainly covalently bonded with lignin via lignin-carbohydrate complex linkages [45], and thus they were inevitably removed along with the lignin during the alkaline ethanol post-treatment. The presence of these carbohydrates in AL may affect the performance of LPF resins prepared from the corresponding lignin fractions to some extent [46]. In the case of the purified EHR, a strong cross-signal of methoxyl was clearly found and other cross-signals of the side-chain linkages disappeared.

The main cross-signals in the aromatic region of the 2D-HSQC spectra of AL are assigned to the aromatic rings of the different lignin units (*p*-hydroxyphenyl (H), guaiacyl (G), and syringyl (S) units). Signals corresponding to *p*-coumarates (PCE) and ferulates (FA) were also observed in this spectrum with relatively low intensities. It should be mentioned that the presence of PCE and FA can also provide potential active sites (unsubstituted 3 or 5 positions of phenolic hydroxyl group) for further utilization, such as LPF resin preparation. In the case of the purified EHR, the cross-signals of S units were very weak, while the signals of G and H units were relatively strong. This indicated that the lignin fraction with a high content of S units was removed as AL during the 4% alcoholic NaOH post-treatment.

**Fig. 6:**
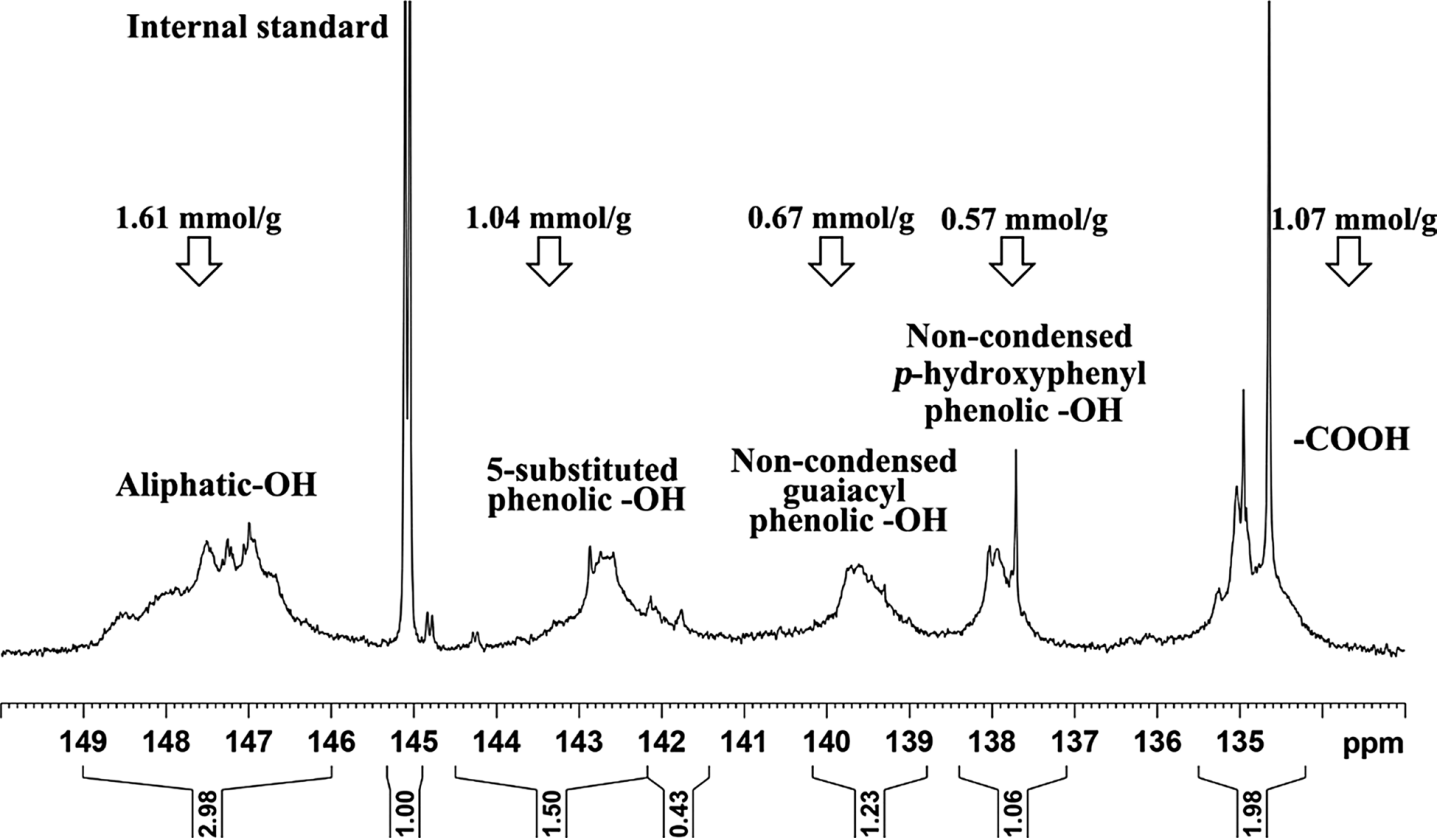
^31^P NMR spectrum of AL

^31^P NMR analysis involving phosphorylation of hydroxyl groups followed by quantitative analysis in the presence of an internal standard allows quantification of all –OH groups present in lignin. For this reason, the content of potential active sites of a lignin for application in LPF adhesive preparation can be obtained using ^31^P NMR analysis [47]. The ^31^P NMR spectrum of AL and the quantitative data on the distribution of the various –OH groups are shown in Fig. 6. The data showed that the content of non-condensed G- and H-type phenolic –OH was 0.67 and 0.57 mmol g^−1^, respectively. The active number calculated based on these data was 1.81 mmol g^−1^, which was slightly higher than that of a technical lignin (1.72 mmol g^−1^) used in our previous study [47]. Therefore, the reactivity of AL could make it as an ideal material for LPF resin preparation. The poor solubility of purified EHR in solution of anhydrous pyridine and deuterated chloroform (1.6:1, v/v) seriously impeded the ^31^P NMR analysis of this sample. Thus, it was not performed in this study.

### LPF resin preparation

The adhesive preparations of LPF resin using AL and EHR were both dark-brown aqueous solutions, and their specific properties are listed in Table 3. Both resins had relatively high solid content (above 50%), which was favorable for forming a continuous bond line between the two cementing limiting surfaces. The viscosities of the ALPF and EHRPF obtained were 987.4 and 766.2 mPa s, respectively. It is well known that the high viscosity of LPF resin will obviously bring problem in the application stage. Fortunately, due to the good water solubility of the synthesized resin products, their viscosity could be adjusted to an acceptable level using water without blemishing the performance of the final products. Similar conclusion has been reported in our previous study [48–50].

**Table 3:** The properties and plywood performances of lignin-phenol-formaldehyde (LPF) resin adhesives

Adhesives	Adhesive properties			Plywood performances	
	pH	Viscosity (mPa s)	Solid content (%)	Bonding strength (MPa)	Formaldehyde emission (mg L^−1^)
ALPF	11.8 (0.6)^c^	987.4 (1.58)	51.3 (0.44)	1.14 (0.11)	0.14 (0.06)
EHRPF	11.3 (0.46)	766.2 (0.90)	53.3 (0.26)	1.01 (0.17)	0.21 (0.02)
GB/T 14732-2006^a^	≥7	≥60	≥35	≥0.7	≤0.5^b^

*ALPF* lignin-phenol-formaldehyde resin adhesive prepared with AL, *EHRPF* lignin-phenol-formaldehyde resin adhesive prepared with EHR

^a^GB/T 14732-2006: Wood adhesives: urea formaldehyde, phenol formaldehyde and melamine formaldehyde resins

^b^This requirement is defined by Chinese National Standard GB/T 9846.3-2004

^c^The value in parenthesis is standard deviation

The bonding strength and formaldehyde emissions of the plywoods prepared with the two LPF resins are also listed in Table 3. It could be observed that the plywoods assembled with the two resins performed differently. Interestingly, the bonding strength of both plywoods (1.14 and 1.04 MPa for samples prepared with ALPF and EHRPF, respectively) met the standard for exterior-grade panels (first grade, >0.7 MPa), and the formaldehyde emissions of the corresponding plywoods were all below 0.5 mg L^−1^ (0.14 and 0.21 mg L^−1^ for samples prepared with ALPF and EHRPF, respectively), meeting E_0_ grade (<0.5 mg L^−1^) plywood requirements under Chinese National Standard GB/T 9846.3-2004 (Plywood-Part 3: General Specification for plywood for general use). It was also found that the performance of the plywoods prepared with either LPF were similar to that of the plywoods prepared with other LPF resins at the same lignin substitution level [50]. Thus, these two LPF resins could be utilized as low-toxicity wood adhesives to prepare both exterior plywood and interior E_0_-grade panels.

The data shown in Table 3 indicated that the LPF resins prepared in this study had good comprehensive performance. However, it should to be noted that a specific performance of the synthesized LPF resin could be adjusted according to the actual requirement. Such adjustment could be realized by changing the mole ratio of phenol to formaldehyde and the content of lignin or lignin-rich residue in the formulation, and consequently it promotes further improvement in the applicability of AL and EHR in LPF resin preparation. In addition, it should also be emphasized that the AL sample used to prepare LPF resins in this study was isolated and purified before resin synthesis. However, neither isolation nor purification of this lignin fraction was necessary for synthesizing LPF resin in a real industrial production scenario. A simple concentration process may be sufficient to obtain lignin solutions with desired solid content. For EHR, desiccation was unnecessary for their utilization in LPF resin preparation since water was also a common component of LPF resin. In fact, the application of EHR in this study inspired the valorization of fermentation residue obtained from bio-ethanol production. The remained yeast in the lignin-rich fermentation residues also contains a large proportion of protein, and may contribute to resin synthesis like cellulase dose. Thus, the lignin-rich fermentation residues can also be appropriate materials for LPF resin preparation.

## Conclusions

An alkaline ethanol NaOH post-treatment under mild conditions was confirmed to be a very effective delignification method, and could effectively promote the cellulose digestibility of the acid-steam-exploded corn stover. It was found that the cellulose digestibility of the acid-steam-exploded corn stover post-treated with 4% alcoholic NaOH reached a maximum of 99.3%, although 10.7% lignin still remained. Except the lignin content of the acid-steam-exploded corn stover, an expansion of cellulose I lattice caused by the alkaline ethanol post-treatment also remarkably contributed to the enzymatic hydrolysis of cellulose. The lignin fractions (AL and EHR) obtained from 4% alcoholic NaOH post-treatment and subsequent enzymatic hydrolysis processes could be converted to high performance LPF resin adhesives, and the plywoods prepared with both LPF adhesives could meet the strength requirements of exterior plywood and limitation value of formaldehyde emissions for E_0_-grade panels. The proposed model for integral valorization of corn stover in this study has demonstrated a good application prospects and promotion of values.

## Methods

### Materials

An acid-steam-exploded corn stover was kindly supplied by a domestic cellulosic ethanol plant at the pilot scale in China. The specific parameters of the corn stover used in the pilot scale plant are not listed here, as they are considered a commercial secret. The acid-steam-exploded materials were washed thoroughly with water to remove all soluble substances, and then the solid residues were collected by filtration and oven-dried at 40 °C for 12 h. The resultant materials, which served as raw materials for further testing in this study, were kept in a desiccator before delignification and analyses. Cellulast 1.5 L (cellulase, 70 FPU g^−1^) and Novozyme 188 (*β*-glucosidase, 240 CBU g^−1^) were purchased from Novozymes Investment Co., Ltd. (Beijing, China). All chemicals used were of analytical grade unless otherwise noted.

### Delignification and enzymatic hydrolysis

The acid-steam-exploded corn stovers were post-treated with 70% ethanol (v/v) containing certain amounts of NaOH (0.05, 0.5, and 4%) at 75 °C for 2 h, respectively, in an airtight container. The solid to liquid ratio was 1: 20 (w/v) for each experiment. All the post-treated materials were filtered and thoroughly washed with distilled water until the wash was colorless and neutral in pH, then dried at 40 °C for 12 h and stored in a desiccator before hydrolysis and analyses. To obtain an extensively delignified sample, an acid sodium chlorite delignification of the acid-steam-exploded corn stover was conducted according to the method reported by Hallac et al. [51]. The acid-steam-exploded corn stover samples obtained from 0.05, 0.5, and 4% alcoholic NaOH post-treatment, and acid sodium chlorite delignified sample were labeled as Sample 1, 2, 3, 4, and 5, respectively. The detailed information of each sample is given in Table 1.

Enzymatic hydrolysis of the acid-steam-exploded corn stover and alkaline ethanol NaOH post-treated samples was performed in 50-mL Erlenmeyer flasks at 50 °C in a shaking air bath at 150 rpm for 72 h. A typical hydrolysis mixture consisted of 0.1 g of sample, 5 mL sodium acetate buffer (50 mM, pH 4.8) supplemented with 20 μL antibiotics tetracycline and 10 μL cycloheximide, and enzyme loading (17.5 FPU cellulase g^−1^ substrate) with a constant ratio of cellulase to *β*-glucosidase (1:2). After hydrolysis, 100 μL sample was taken from the reaction mixture at certain intervals, deactivated in boiling water for 5 min, then centrifuged for 10 min at 10,000 rpm, and stored at −20 °C. The released glucose was also analyzed by high-performance anion exchange chromatography (HPAEC) system (Dionex ICS3000, US) equipped with a CarboPac PA 100 analytical column. A gradient solvent system was used as the mobile phase, consisting of ultra-pure water, NaOH (0.25 M) and NaAc (1 M). The flow rate was adjusted to 0.4 mL min^−1^ and the volume injection was 20 μL with a column temperature set at 30 °C. All measurements were carried out in duplicate.

**Fig. 7:**
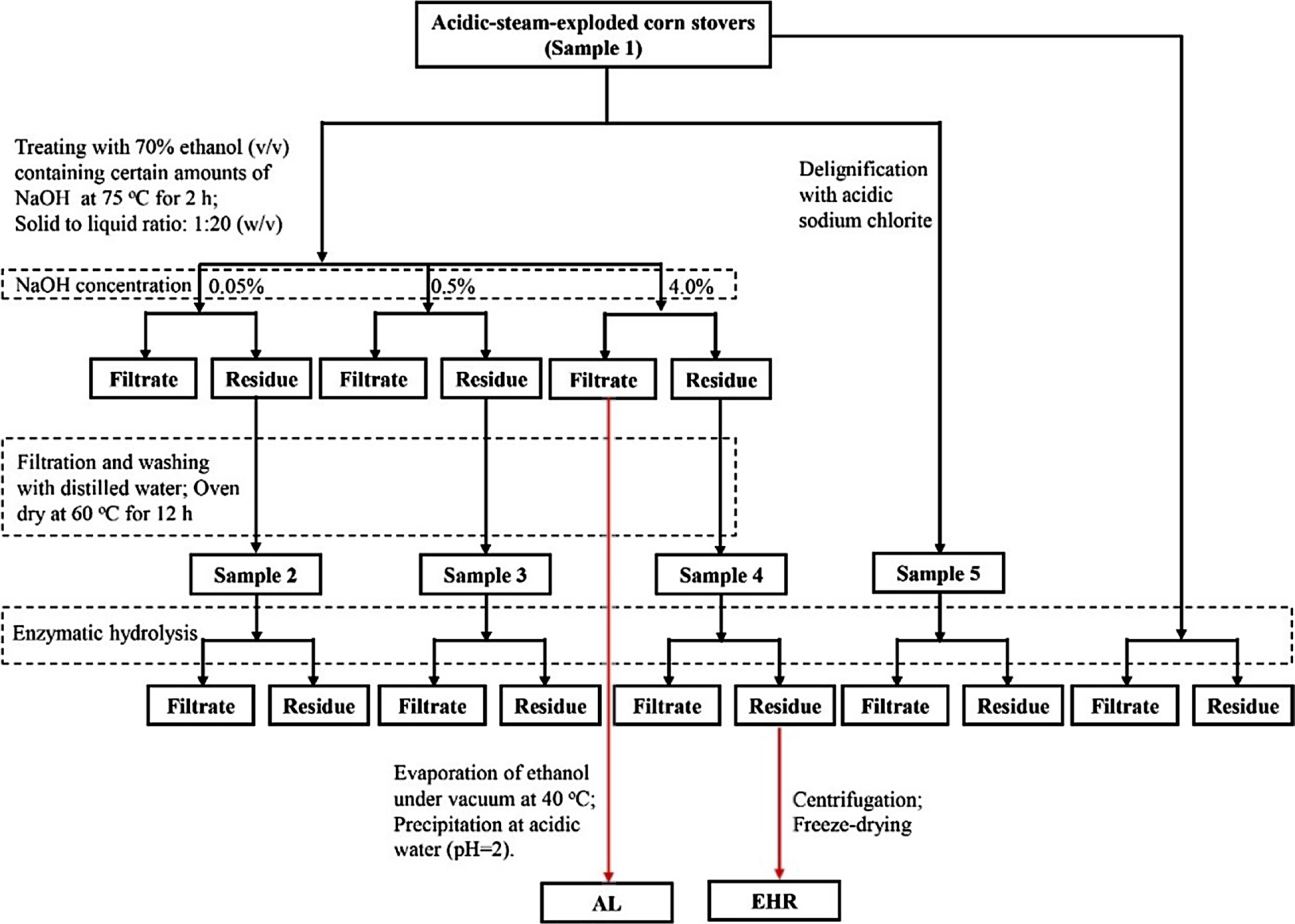
Brief schematics for delignification and subsequent enzymatic hydrolysis processes

The lignin fraction in the effluent from the 4% alcoholic NaOH post-treatment (AL) was collected through acid (pH = 2) precipitation after evaporating ethanol. The lignin-rich residue remained after the enzymatic hydrolysis of the 4% alcoholic NaOH post-treated acid-steam-exploded corn stover (EHR) was also collected via centrifugation followed by freeze-drying. For the 2D HSQC NMR analysis, part of the lignin-rich residue remained after enzymatic hydrolysis was thoroughly washed with hot water to remove residual cellulase, and a purified EHR was recovered. Brief schematics for delignification and subsequent enzymatic hydrolysis processes are shown in Fig. 7.

### Compositional analysis

The chemical compositions of the untreated corn stover, the acid-steam-exploded corn stover and the alkaline ethanol post-treated samples, AL as well as EHR were determined according to the National Renewable Energy Laboratory (NREL) protocol [52]. The total lignin content was the summation of acid-soluble lignin and acid-insoluble lignin. The sugars were quantified on a HPAEC system (Dionex ICS3000, US) equipped with a pulsed amperometric detector, an AS50 autosampler, a Carbopac TM PA-20 column (4 × 250 mm, Dionex), and a guard PA-20 column (3 × 30 mm, Dionex) according to a previously published method [41]. The sugar fractions were separated in a 5 mM NaOH isocratic (carbonate free and purged with nitrogen) condition for 20 min, followed by a 0–75 mM NaAc gradient in 5 mM NaOH for 15 min. Then the columns were washed with 200 mM NaOH to remove carbonate for 10 min, and followed a 5 min elution with 5 mM NaOH to re-equilibrate the column before the next injection. The total analysis time was 50 min and the flow rate was 0.4 mL min^−1^. The analysis of sugar composition in the present study was run in duplicate.

### Characterization

The acid-steam-exploded corn stover and alkaline ethanol post-treated samples were evaluated by FT-IR spectroscopy, which were obtained ranging from 4000 to 675 cm^−1^ under ATR mode using a Thermo Scientific Nicolet iN10 FTIR spectrometer (Thermo Nicolet Corporation, Madison, WI, USA) equipped with a liquid nitrogen-cooled MCT detector at room temperature. The solid-state CP/MAS ^13^C-NMR experiments were performed at 100 MHz using a Bruker AV-III 400 M spectrometer (Bruker, Karlsruhe, Germany). Each sample was packed in a 4 mm zirconia rotor, then measured with a CP pulse program with 1 ms match time and 2 s delay between transients at spinning rate of 5 kHz. X-ray diffraction patterns of the samples were recorded on an XRD-6000 instrument (Shimadzu, Japan) ranging from 5 to 35° of 2θ using a goniometer at a scanning speed of 2° min^−1^. The scan was performed with Ni-filtered Cu Kα radiation (λ = 1.54 Å) at 40 kV and 30 mA, and the crystallinity index (*CrI*) of the samples was calculated as the ratio of the area of the resolved crystalline peaks to the total area of a diffraction profile for 5–35° based on the X-ray diffraction curves of various samples. Structural features of the AL were investigated using 2D HSQC spectroscopy according to a method reported by a previous paper [53]. The 2D HSQC spectra were recorded at 25 °C on a Bruker AVIII 400 MHz spectrometer. 80 mg of lignin sample was dissolved in 0.5 mL of DMSO-*d*_6_. The central solvent peak at *δ*_C_/*δ*_H_ 39.5/2.49 was used as an internal reference. The spectral widths were 5000 and 20,000 Hz for the ^1^H and ^13^C dimensions, respectively. The number of collected complex points was 1024 for the ^1^H dimension with a recycle delay of 1.5 s. The number of transients was 64, and 256 time increments were always recorded in the ^13^C dimension. The ^1^ *J*_CH_ used was 145 Hz. Prior to Fourier transformation, the data matrices were zero filled to 1024 points in the ^13^C dimension. The hydroxyl groups of AL were quantitatively analyzed by ^31^P NMR technique. ^31^P NMR spectrum was acquired after the reaction of lignin with 2-chloro-4, 4, 5, 5-tetramethyl-1, 3, 2-dioxaphospholane (TMDP). 20 mg of lignin sample was dissolved in 500 μL anhydrous pyridine and deuterated chloroform (1.6:1, v/v) under stirring. Cholesterol and chromium (III) acetylacetonate were used as internal standard and relaxation reagent, respectively. TMDP was used as phosphorylating reagent. The parameters used in quantitative ^31^P NMR experiment were listed as follows: 30° pulse angle, 2 s relaxation delay (*d*_1_), 64 K data points and 1024 scans. The poor solubility of EHR (or purified EHR) in the solution of anhydrous pyridine and deuterated chloroform (1.6:1, v/v) seriously impeded the ^31^P NMR analysis of this sample. Thus, it was not conducted in this study.

### Lignin-phenol-formaldehyde resin preparation

The lignin fractions (AL and EHR) obtained from the 4% alcoholic NaOH integral biorefinery process were used to synthesize LPF resins (ALPF and EHRPF resins) using the same procedure as our previously reported study [50]. The substitution rate of lignin to phenol was set as 40% (w/w) and the mole ratio of the phenol to formaldehyde was 1:1.8 for LPF resin preparation. Phenol and lignin were mixed in a three-necked flask followed by the addition of appropriate amount of NaOH solution (30 wt%). The mixture was heated to 90 °C and held at the temperature for 1 h. The temperature of the mixture was then dropped to 80 °C, and 70% of the total formaldehyde and NaOH solution (30 wt%) was added. This mixture was kept at 80 °C for 1 h. In sequence, the remaining 30% of the total formaldehyde and NaOH solution (30 wt%) was added, and that mixture was kept at 80 °C for 1 h. Finally, the temperature of the mixture was decreased to 65 °C, and the solution of NaOH (30 wt%) and urea (5% of the total weight of phenol and lignin) was added. This mixture was held at 65 °C for 30 min. When the reaction was complete, it was rapidly cooled to 40 °C to yield the LPF resin. The pH, viscosity, and nonvolatile content of the LPF resins were determined in accordance with the Chinese National Standard (GB/T 14704-2006: Testing methods for wood adhesives and their resins). The synthesized LPF resins were used as wood adhesive to prepare three-layer poplar plywoods (400 * 400 * 4.5 mm) by coating core poplar veneer with a 150 g m^−2^ adhesive on each side, then hot-pressing the plywoods at 145 °C under 1.0 MPa for 7 min. The bonding strength and formaldehyde emissions of the plywoods were measured according to the Chinese National Standard (GB/T 17657-2013: Testing methods of evaluating the properties of wood-based panels and surface decorated wood-based panels).

## Abbreviations

ASE: acid steam explosion
NaOH: sodium hydroxide
AL: the lignin fraction released during the 4% alcoholic NaOH post-treatment
EHR: lignin-rich residue remained after the enzymatic hydrolysis of the 4% alcoholic NaOH post-treated acid-steam-exploded corn stover
LPF: lignin-phenol-formaldehyde
FT-IR: Fourier transform infrared
CP/MAS ^13^C-NMR: cross polarization/magic angle spinning ^13^C nuclear magnetic resonance
XRD: X-ray diffraction
*CrI*: crystallinity index
HPAEC: high-performance anion exchange chromatography
NREL: National Renewable Energy Laboratory
2D HSQC: two dimensional heteronuclear single-quantum correlation
ALPF resin: LPF resin prepared with AL
EHRPF: LPF resin prepared with EHR
